# Dimeric SecA Couples the Preprotein Translocation in an Asymmetric Manner

**DOI:** 10.1371/journal.pone.0016498

**Published:** 2011-01-27

**Authors:** Ying Tang, Xijiang Pan, Yong Chen, Phang C. Tai, Sen-Fang Sui

**Affiliations:** 1 State Key Laboratory of Biomembrane and Membrane Biotechnology, Center for Structural Biology, School of Life Sciences, Tsinghua University, Beijing, China; 2 Department of Biology, Georgia State University, Atlanta, Georgia, United States of America; University of South Florida College of Medicine, United States of America

## Abstract

The Sec translocase mediates the post-translational translocation of a number of preproteins through the inner membrane in bacteria. In the initiatory translocation step, SecB targets the preprotein to the translocase by specific interaction with its receptor SecA. The latter is the ATPase of Sec translocase which mediates the post-translational translocation of preprotein through the protein-conducting channel SecYEG in the bacterial inner membrane. We examined the structures of *Escherichia coli* Sec intermediates in solution as visualized by negatively stained electron microscopy in order to probe the oligomeric states of SecA during this process. The symmetric interaction pattern between the SecA dimer and SecB becomes asymmetric in the presence of proOmpA, and one of the SecA protomers predominantly binds to SecB/proOmpA. Our results suggest that during preprotein translocation, the two SecA protomers are different in structure and may play different roles.

## Introduction

The general secretory pathway in bacteria involves a multipartite protein machine, Sec translocase, which is responsible for transferring unfolded newly synthesized polypeptides across the inner membrane [1]. The nascent polypeptides are captured by the SecB chaperone in a translocation-competent state after they are released from the ribosome [2]. Then, the complex is targeted to SecA, the translocation motor. SecA couples the stepwise translocation of preprotein across the SecYEG channel with the expenditure of metabolic energy provided by consecutive ATP binding and hydrolysis cycles [3]. *In vitro* translocation assays show that the ATPase activity of SecA is required for the transmembrane translocation of the amino terminus of the preprotein, including the signal sequence [4], [5], [6]. In the absence of a transmembrane proton-motive force, SecA is also necessary for the translocation of the downstream carboxyl terminus [4].

In solution, SecA exists in a dynamic equilibrium between the monomeric and dimeric forms with a K_d_ of approximately 1 µM [7]. Increased temperature or protein concentration and reduced ionic strength stabilize the SecA dimer [7]. SecA can bind *in vivo* to SecYEG, SecB, the preproteins of each, its own mRNA, acidic phospholipids, nucleotides, and divalent cations such as Mg^2+^ and Zn^2+^, and these interactions induce conformational changes in the protein, making the oligomeric nature of SecA more complicated [8]. The oligomeric state of SecA during preprotein translocation, information that is crucial to understanding the mechanism of Sec translocase, remains controversial.

The components of the Sec machinery interact with each other and function together to accomplish preprotein translocation through various translocation intermediate complexes. Previous studies have shown that SecB forms tetramers in solution [9]. How SecA interacts and works together with SecB to deliver preproteins to the channel is a key to understanding the mechanism of translocation, but remains unexplained. The structures of the intermediate translocation complexes should provide direct evidence of the oligomeric state of SecA and help to elucidate the mechanism of Sec translocase.

Electron microscopy and single-particle image analysis allow the direct visualization of macromolecular protein complexes in solution. In this study, we find that SecA and SecB can form a relatively stable complex, and SecA, SecB, preprotein (here we use proOmpA) can also form a ternary complex which is suitable for EM study. We use single-particle electron microscopy to investigate the structure of two Sec protein complexes in solution, SecA/SecB and SecA/SecB/proOmpA. Our results indicate that in solution, although SecA binds to proOmpA and SecB as a homo-dimer, only one of the SecA protomers is predominantly involved in the interactions with the latter two molecules. Our work suggests that dimeric SecA couples the preprotein translocation in an asymmetric manner.

## Results

### Dimeric SecA can form a symmetric complex with tetrameric SecB in solution

**Figure 1:**
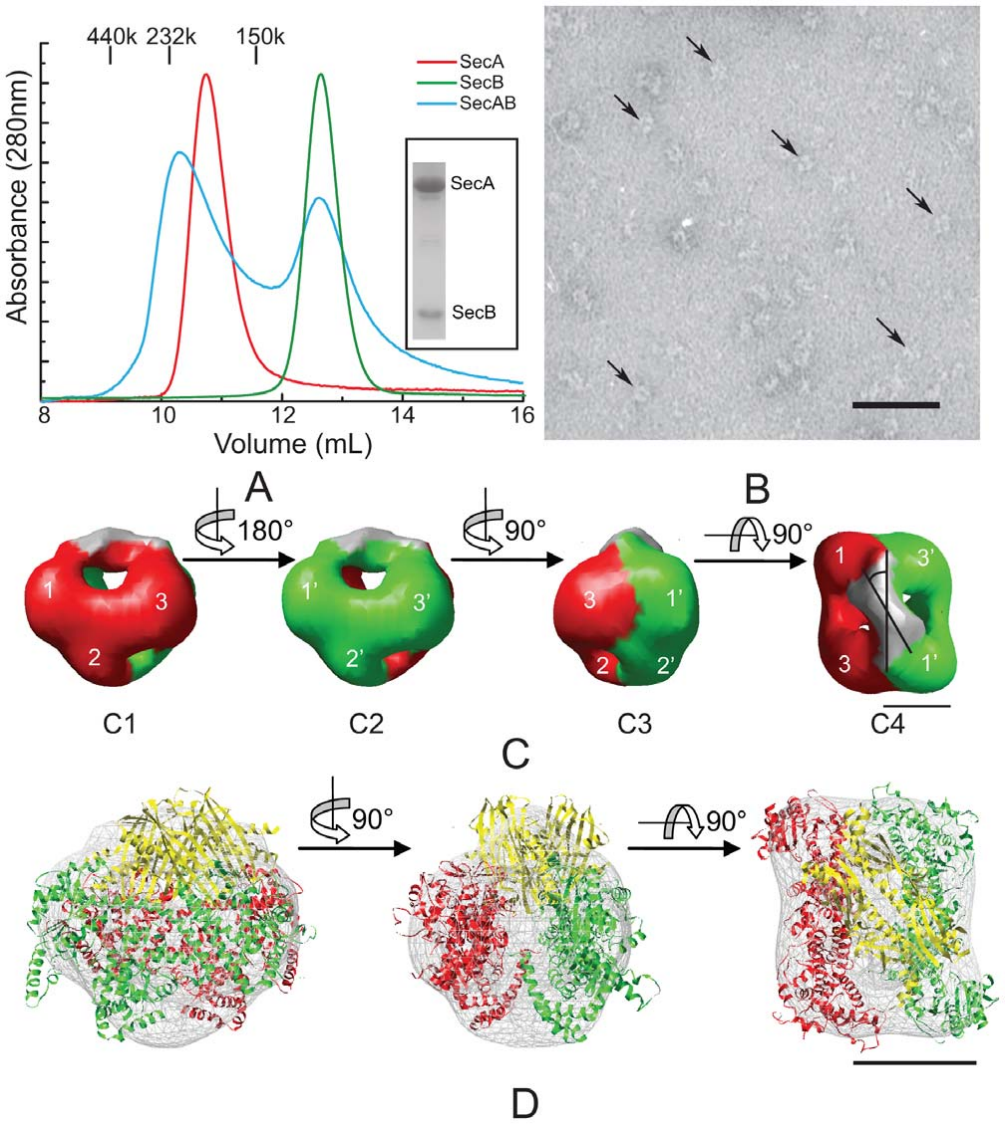
Complex formation between SecA and SecB. (A) Absorbance profiles of SecA and SecB complexes resolved by size-exclusion chromatography. Red line: SecA alone (6 µM monomer). Green line: SecB alone (40 µM monomer). Cyan line: SecA monomer (3 µM) and SecB monomer (30 µM). Marker protein: aldolase (150 kDa), catalase (232 kDa), and ferritin (440 kDa). The inserted SDS-PAGE panel shows the contents for one fraction (from 10.0 to 10.3 mL) of the cyan line after TCA precipitation with Coomassie Blue staining. (B) EM image of negatively stained SecA/SecB complex. Distinct particles are indicated by arrows. The scale bar represents 50 nm. (C) Surface representation of the 3D reconstruction of the SecA/SecB complex (C1, C2, C3, C4). The two SecA protomers were rendered with different colors: green and red. The three domains in each SecA protomer are designated 1, 2, and 3, and 1′, 2′, and 3′. Four views are shown: side (C1, C2, C3) and top (C4). Each view was obtained after a 90° rotation operation around the axis as shown between these views. (D) Docking of the SecB (PDB ID: 1QYN) and closed *E. coli* SecA X-ray crystal structures (ecSecA-closed) into the EM 3D map. The three views are the same views as the C2–C4 respectively. The X-ray crystal structure of each SecA is colored green or red. The X-ray crystal structure of SecB is rendered in yellow. The contour EM density map of the SecA/SecB complex is represented by grey hatch markers. The scale bars represent 5 nm in (C) and (D).

The interaction between SecA and SecB plays an important role in the process of preprotein targeting to the translocation channel [10]. We monitored the formation of the SecA/SecB complex by size-exclusion chromatography (Figure 1A). Since it has been shown that the stability of the complex is sensitive to the salt concentration [11], SecA and SecB were incubated in buffers of low ionic strength. The SecA dimer concentration was much lower than that of the SecB tetramer to ensure that most of SecA would form a complex with SecB. This condition facilitated the separation of the SecA/SecB complex from SecB alone. When a mixture of SecA and SecB incubated at 4°C for 1 h was applied to the column, two peaks appeared. The first peak corresponds to an estimated molecular weight of 230 kDa, consistent with the sum of dimeric form of SecA and a tetramer of SecB (calculated mw 272 kDa). This result was verified by SDS-PAGE (Figure 1A). The second peak eluted at the same position as SecB alone.

The fractions collected from the size-exclusion chromatography containing the SecA/SecB complex were immediately applied to electron microscopy grids and fixed by negative staining. The negatively stained specimens demonstrated well-preserved particles with characteristic structures (Figure 1B). We reconstructed the 3D structure of the SecA/SecB complex at 18 Å resolution by angular reconstruction. There is an obvious two-fold symmetry in the density map (Figure S1A), therefore, we imposed C2 symmetry on the final 3D model. The model shows a wedge-shaped structure composed of two antiparallel subunits with a region of elongated density at the top (Figure 1C). Each of the antiparallel subunits shows a prominent comma-shaped structure with three domains, designated domains 1, 2, and 3 (Figure 1C). The region of elongated density covers the cavity surrounded by domains 3 and 1 from the two separate antiparallel subunits.

We then docked the *E. coli* SecA (ecSecA-closed) [12], [13] and *E. coli* SecB X-ray crystal structures [14] into the EM density map to identify the localization of the molecules and domains in this complex (Figure 1D). We used the automatic docking software SITUS [15] to perform the docking simulation. Two SecA monomers dock into the wedge-shaped part of the model. As we identified previously by cryo-EM [16], domain 1 corresponds to the preprotein binding domain (PBD) and the C-terminal domain, domain 2 corresponds to the nucleotide binding domain 1 (NBD1), and domain 3 corresponds to the nucleotide binding domain 2 (NBD2). The PBD and C-terminal domains could not be clearly separated in the density map at the current resolution. With the majority of the 3D density map filled by the SecA atomic model, we were able to calculate the difference map between the reconstruction and the low-pass filtered volume from docked atomic models of the SecA dimer. The SecB tetramer atomic model was then docked into the difference density map using the ‘qrange’ program of the SITUS package [15]. One SecB tetramer can fit into this difference density map. The shrink effect of negative staining may be the reason why the density map is a little smaller than the atomic model.

Using the COLORES [15] program, we found that the closed conformation of *E. coli* SecA can fit relatively better into the density map compared with the open conformation of SecA (ecSecA-open [17])(Figure S3). If we use the open conformation of *E. coli* SecA to perform the docking procedure, the PBD will protrude from the domain 1 of the EM density map (Figure S3B).

This clearly shows that one SecA dimer interacts with one SecB tetramer to form a complex in solution. The long axis of the SecB tetramer is tilted 30° from the long axis of the SecA dimer, and their two-fold axes superimpose in the complex (Figure 1 C4).

### The presence of proOmpA induces an asymmetric rearrangement of dimeric SecA in the SecA/proOmpA/SecB ternary complex

**Figure 2:**
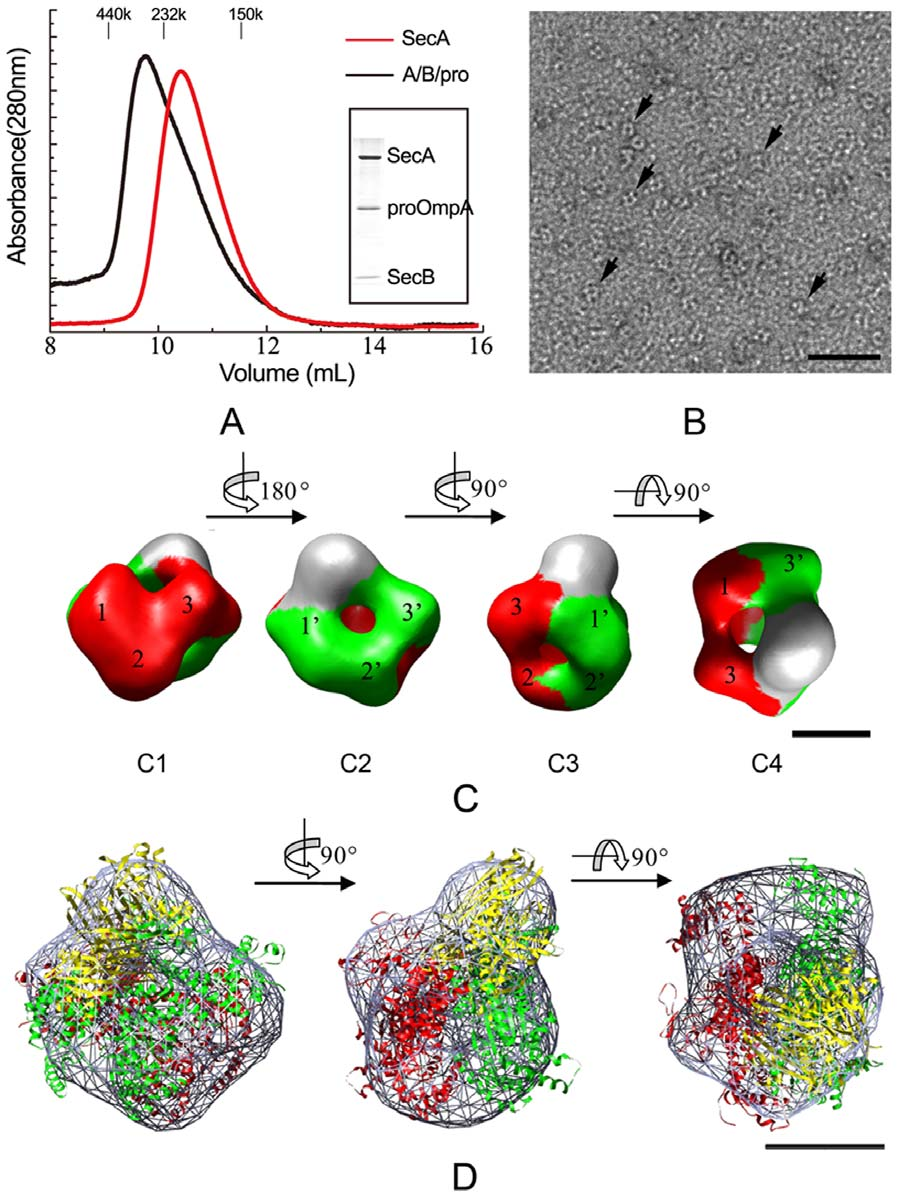
Complex formation of SecA, SecB and proOmpA. (A) Absorbance profiles of complexes of SecA, SecB, and proOmpA resolved by size-exclusion chromatography. The samples applied were 3 µM SecA monomer (red) and a mixture of 3 µM SecA monomer and 2 µM SecB/proOmpA (black). Fractions (300 µL) were collected and analyzed by SDS-PAGE with Coomassie Blue staining. The insert panel shows the result for the fraction with the highest absorbance at 280 nm. (B) Electron microscopy image of negatively stained SecA/proOmpA/SecB complexes. Some distinct particles are indicated by arrows. The scale bar represents 50 nm. (C) Surface representation of the 3D reconstruction of the SecA/proOmpA/SecB complex (C1, C2, C3, C4). The two SecA protomers are rendered in green and red. The unstained domain represents SecB/proOmpA. The three domains in each SecA protomer are designated 1, 2, and 3, and 1′, 2′, and 3′. Four views are shown of the surface representation: side view (C1, C2, C3) and top view (C4). Each view was obtained after the rotation operation around the axis as shown between these views. (D) Docking of the *E. coli* SecA (ecSecA-open) and SecB (PDB: 1QYN) X-ray structures into the EM 3D map. The three views are the same views as the C2–C4 respectively. The X-ray crystal structure of each *E. coli* SecA is colored green or red. The X-ray crystal structure of SecB is rendered in yellow. The contour of the EM density map of the SecA/proOmpA/SecB complex is represented with grey hatch markers. The scale bars represent 5 nm in (C) and (D).

The targeting of preprotein and SecB complex to SecA initiates Sec translocation. To investigate the mechanism of preprotein delivery and the oligomeric state of SecA during this process, we examined the structure of the SecA/proOmpA/SecB ternary complex by single-particle EM. We assembled stable complexes of proOmpA and SecB by rapid dilution as described by Lecker et al. [18] and incubated SecA with the purified proOmpA/SecB complex at 4°C for 1 h. The mixture was then applied to the size-exclusion column. Only one peak containing SecA, SecB, and proOmpA appeared at the observed molecular weight of 300 kDa (Figure 2A). This indicated that dimeric SecA was bound to one SecB tetramer with one proOmpA in the complex (307 kDa in total). Therefore, dimeric SecA, proOmpA, and SecB can form a stable complex. Using *in vitro* assays, we further verified that the SecA/proOmpA/SecB complex has translocation activity with SecYEG proteoliposomes (data not shown).

The SecA/proOmpA/SecB complex appears as heterogeneous particles in negatively stained electron microscopy which is maybe due to the disassociation of SecA and SecB/proOmpA (Figure 2B). Manual particle picking was carefully performed and a multi-model-refinement was also carried on to deal with this heterogeneous problem (Figure S2A, S2B). Finally we obtained the 3D reconstruction of the complex at 24 Å from 2,704 particles after 12 iterations of refinement (Figures 2C and S2). The reconstructed model also shows a wedge-shaped structure composed of two subunits with three domains and a region of elongated density on top (Figure 2C), but does not show two-fold symmetry as in the SecA/SecB complex. Since the unfolded proOmpA wraps around the chaperone [9], in this ternary complex SecB is the scaffold of the SecB/proOmpA complex. The atomic models of the *E. coli* SecA and SecB were again docked into the EM density map (Figure 2D, and Figure S4). Although SecA still docks as a dimer in this complex, the two SecA protomers are no longer in a symmetric arrangement. One SecA protomer (in green in Figure 2C and Figure S4) fits better with the open structure of SecA (ecSecA-open, 17) compared with the closed structure. As for the second protomer (in red), we cannot distinguish whether it is in the open or closed conformation. The interaction interfaces between SecB and the two SecA protomers are obviously different. For the former (in green), domains 1 and 3 both interact directly with the SecB/proOmpA complex; whereas for the latter (in red), only domain 3 is involved in the interaction (Figure 2 C4). Thus, the SecB/proOmpA complex bound predominantly to only one of the two SecA protomers. This suggests that the involvement of proOmpA changed the nature of the interaction of SecB/SecA and the two SecA protomers adjusted themselves through asymmetric rearrangement to meet the requirements of the next step of preprotein translocation.

## Discussion

Whether SecA functions as a dimer or a monomer during preprotein translocation is controversial. We studied the structures of two Sec protein complexes in solution involved in tandem translocation steps by electron microscopy, providing direct structural evidence of the oligomeric state of SecA in the initiation of preprotein translocation. Our data show that dimeric SecA may mediate translocation of the preprotein in an asymmetric manner. Each protomer of the SecA dimer undergoes different conformational changes upon preprotein targeting.

The most notable characteristic of the SecA/proOmpA/SecB complex is its asymmetry, which is in accordance with previous results [19], suggesting that the two SecA protomers have different binding affinities for SecB/proOmpA and only one of the SecA protomers predominantly binds with SecB/proOmpA. Interestingly, data emerging from studies of the crystal structures of the SecA proteins show that the PBD can adopt two different conformations: open and closed. In the closed conformation, PBD interacts with the C domain, forming a compact structure [20]. However, in the open conformation, PBD undergoes a ∼60° rigid-body rotation and exposes most of its surface to the solvent [12], [17]. We have determined the structure of SecA in the apo state, demonstrating that the ligand-free SecA is a dimer in the closed conformation in solution [16]. This type of conformation is not affected by the binding of SecB (Figure 1A). However, our results suggested the SecA protomer that predominantly binds with SecB/proOmpA in the SecA/proOmpA/SecB ternary complex is in the open state (Figure S4). These results are in accordance with previous reports [13]. In the open state, the groove of SecA is exposed, allowing the recognition of signal sequences, and is conducive to the preprotein binding [13].

The asymmetric binding of SecB/proOmpA led to a change in the interface between the two SecA protomers, which may cause a change in the biochemical properties of SecA. This might be the reason for the progressively weaker dimer cross-linking with increasing synthetic signal sequence concentration [21]. This kind of asymmetric binding between SecA, proOmpA, and SecB may also contribute to the dissociation of SecB at the beginning of preprotein translocation.

## Materials and Methods

### Materials

SecA [22], His_6_-tagged SecB [23], proOmpA [24] were purified as previously described. Superdex G200 was from Amersham Pharmacia. Nanosep centrifugal Devices were from PALL. All others are reagents grade, and were purchased from commercial sources. Protein samples were analyzed by SDS-PAGE using 12–15% acrylamide gels.

### Complex formation and size-exclusion chromatography

For SecA/SecB complex, SecA monomer (3 µM) and SecB monomer (30 µM) were incubated at 4°C in buffer A (20 mM HEPES-KOH, 30 mM KCl, 1 mM DTT, pH 7.4) for 1 hour. For SecA/proOmpA/SecB complex, proOmpA (300 µM) in 6 M urea was added to 30 µM His_6_-tagged SecB tetramer in buffer A by rapid dilution. The protein mixture was further diluted with buffer A and concentrated by ultrafiltration until the urea concentration was less than 5 mM. The final concentration of SecB/proOmpA was about 30 µM. SecA monomer (3 µM) and SecB/proOmpA (2 µM) were incubated at 4°C for 1 hour. 100 µL of protein sample was loaded onto a Superdex G200 analytical size-exclusion column equilibrated with buffer A. Separation was carried out at 4°C at 0.5 mL/min, and absorbance was monitored at 280 nm. The molecular weights of the proteins were estimated by comparing the retention times of a set of protein standards of known molecular weight: Aldolase (150 kDa), Catalase (232 kDa), Ferritin (440 kDa).

### EM and 3D reconstruction

For negative staining, 5 µL sample was applied to a hydrophilic carbon-coated EM grid for 1 min, and then stained with 1% uranyl acetate (w/v) for 1 min. Specimens were examined in a Phillips CM120 microscope operated at 100 kV. Images were recorded on Kodak SO-163 films at 52,000× magnification under low-dose conditions. Following development in full-strength D19 for 6 minutes, selected images were digitized with a Nikon Coolscan 9000ED scanner at a step size of 12.7 µm/pixel, yielding a pixel size of 2.44 Å on the specimen scale.

For 3D reconstruction, the procedure followed the EMAN standard protocols [25]. To be briefly, 1872 particles for SecA/SecB complex and 3526 for SecA/proOmpA/SecB were picked out using BOXER program. After initially centered and rotationally aligned, selected particles were classified and 7–9 class averages were selected using StartAny to generate an initial 3D model and then refined for 12 rounds until the 3D models were convergent. For the SecA/proOmpA/SecB complex, another round of multi-model-refinement was carried on, using low pass filtered cryo-EM SecA 3D model [16] and the model got in the previous step as initial models. About 30% of the raw particles were assigned to SecA (Figure S2B), some of the particles shows a C2 symmetry which accords with our previous study [16]. The rest 2704 particles were used in the final refinement to get the SecA/proOmpA/SecB 3D structure. The final resolutions of the 3D reconstructions were estimated from the Fourier shell correlation (FSC) curve using the FSC-0.5 cut-off criterion.

### Docking analysis

3D visualization was performed using Chimera [26]. Rigid-body fitting of SecA and SecB into the model of SecA/SecB complex was performed using Situs described in the text [15], while fitting into the model of SecA/SecB/proOmpA complex was performed in Chimera using “Fit model in map”. The SecA crystal structure in open state (ecSecA-open) is a kindly gift from Dr. Petratos which is the ecSecA structure (2FSF) with the modelled PBD [17]. And the SecA in the closed state structure (ecSecA-closed) is generated from the open state one by rotating the PBD about 60° [12], [13].

## Supporting Information

Figure S1 **Evaluation of 3D reconstruction of SecA/SecB.** (A) Selected class averages for symmetry analysis. (B) A gallery of class averages matching the projections of the SecA/SecB 3D model. The odd columns are projections of this 3D model. The even columns are class averages. (C) A plot representing the Euler angle distribution of classified particles within the asymmetric triangle. The brightness of each point indicates the number of particles used in the class average in that orientation on a log scale. The relatively uniform distribution indicates that there was no missing cone in the Fourier space. (D) Resolution curve of the 3D reconstruction. The resolution calculated from Fourier shell correlations was 18 Å. (E) and (F) The SecA/SecB 3D model refined before (E) and after (F) 2-fold symmetry imposed. Each view is obtained after the 90° rotation operation around the horizontal axis as shown between these views. The scale bar in (A), (E) and (F) represents 5 nm.
(TIF)

Figure S2 **Evaluation of 3D reconstruction of SecA/proOmpA/SecB.** (A) A gallery of selected raw particles from EM images. (B) A gallery of particles assigned to model SecA in the multi-model-refinement. (C) A gallery of class averages matching the projections of the SecA/proOmpA/SecB 3D model. The odd columns are projections of this 3D model. The even columns are class averages. The scale bar in A–C is 5nm. (D)A plot representing the Euler angle distribution of classified particles within the asymmetric triangle. The brightness of each point indicates the number of particles used in the class average in that orientation on a log scale. The relatively uniform distribution indicates that there was no missing cone in the Fourier space. (E)Resolution curve of the 3D reconstruction. The resolution calculated from Fourier shell correlations (FSC) was 24 Å.
(TIF)

Figure S3 **Docking of the 3D EM map of the SecA/SecB complex with the X-ray crystal structures of ecSecA-closed (A) or the ecSecA-open (B), and the X-ray crystal structure of SecB (PDB ID: 1QYN), shown in stereo diagrams.** Two SecA protomers are rendered in different colors, green and red. For each SecA protomer, the PBD is in deepskyblue. The SecB tetramer is rendered in yellow. The scale bar represents 5 nm.
(TIF)

Figure S4 **Docking of the X-ray structures of** ***E.coli*** **SecA into the 3D EM map of the SecA/proOmpA/SecB complex, shown in stereo diagrams.** One SecA protomer, predominantly binding with SecB/proOmpA, is rendered in green with its PBD domain in deepskyblue. The other one is rendered in red. The red SecA protomer is in the open state (ecSecA-open). (A) The green SecA protomer is in the open state. (B) The green SecA protomer is in the closed state (ecSecA-closed). The scale bar represents 5 nm.
(TIF)
