# Supplementary figures and images for: Dimeric SecA Couples the Preprotein Translocation in an Asymmetric Manner
Source: PLoS One. 2011 Jan 27;6(1):e16498. doi: 10.1371/journal.pone.0016498 (PMC3029384; doi:10.1371/journal.pone.0016498)

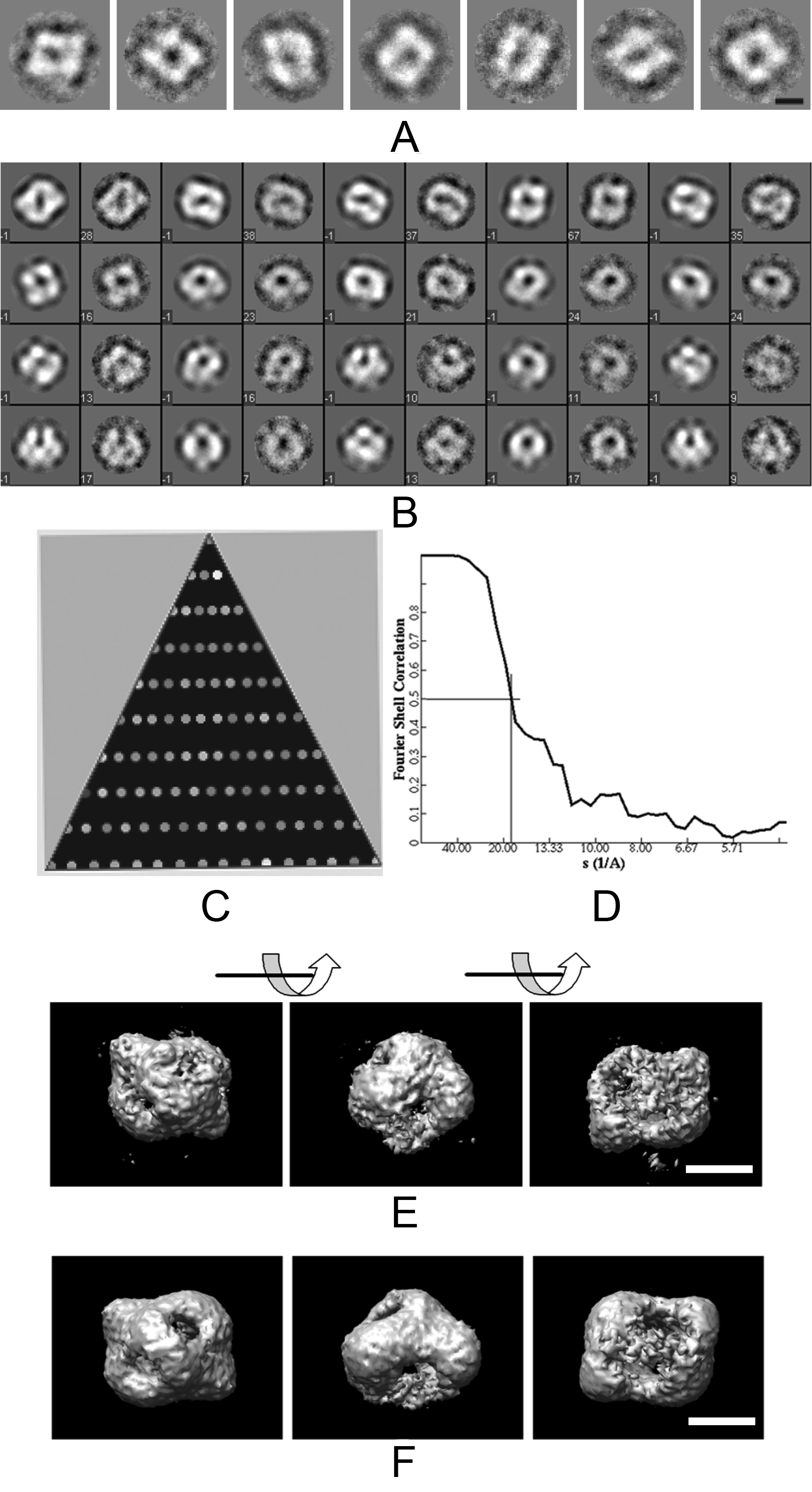

Supplement: Figure S1 — Evaluation of 3D reconstruction of SecA/SecB. (A) Selected class averages for symmetry analysis. (B) A gallery of class averages matching the projections of the SecA/SecB 3D model. The odd columns are projections of this 3D model. The even columns are class averages. (C) A plot representing the Euler angle distribution of classified particles within the asymmetric triangle. The brightness of each point indicates the number of particles used in the class average in that orientation on a log scale. The relatively uniform distribution indicates that there was no missing cone in the Fourier space. (D) Resolution curve of the 3D reconstruction. The resolution calculated from Fourier shell correlations was 18 Å. (E) and (F) The SecA/SecB 3D model refined before (E) and after (F) 2-fold symmetry imposed. Each view is obtained after the 90° rotation operation around the horizontal axis as shown between these views. The scale bar in (A), (E) and (F) represents 5 nm. (TIF) [file pone.0016498.s001.tif]

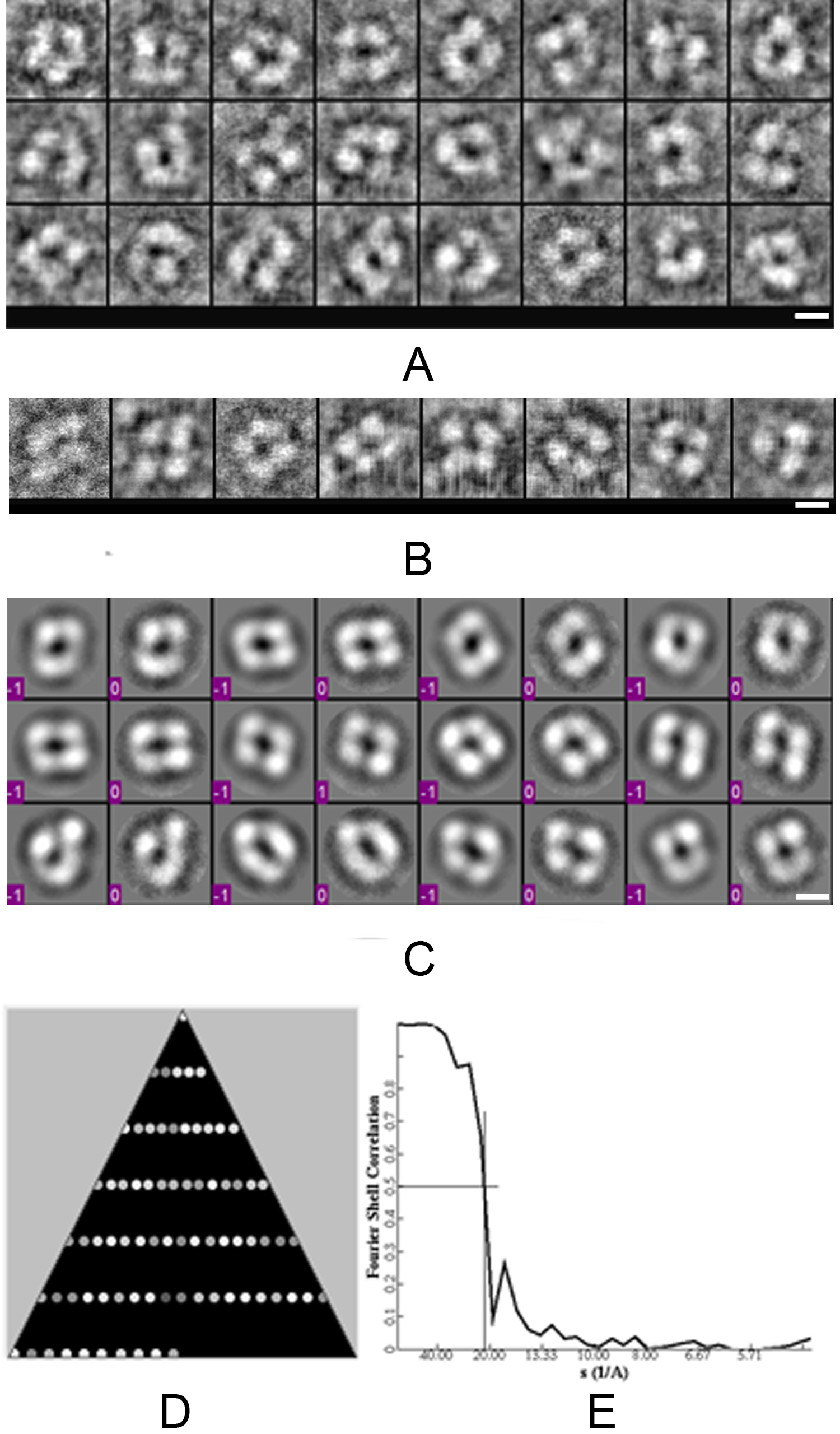

Supplement: Figure S2 — Evaluation of 3D reconstruction of SecA/proOmpA/SecB. (A) A gallery of selected raw particles from EM images. (B) A gallery of particles assigned to model SecA in the multi-model-refinement. (C) A gallery of class averages matching the projections of the SecA/proOmpA/SecB 3D model. The odd columns are projections of this 3D model. The even columns are class averages. The scale bar in A–C is 5nm. (D)A plot representing the Euler angle distribution of classified particles within the asymmetric triangle. The brightness of each point indicates the number of particles used in the class average in that orientation on a log scale. The relatively uniform distribution indicates that there was no missing cone in the Fourier space. (E)Resolution curve of the 3D reconstruction. The resolution calculated from Fourier shell correlations (FSC) was 24 Å. (TIF) [file pone.0016498.s002.tif]

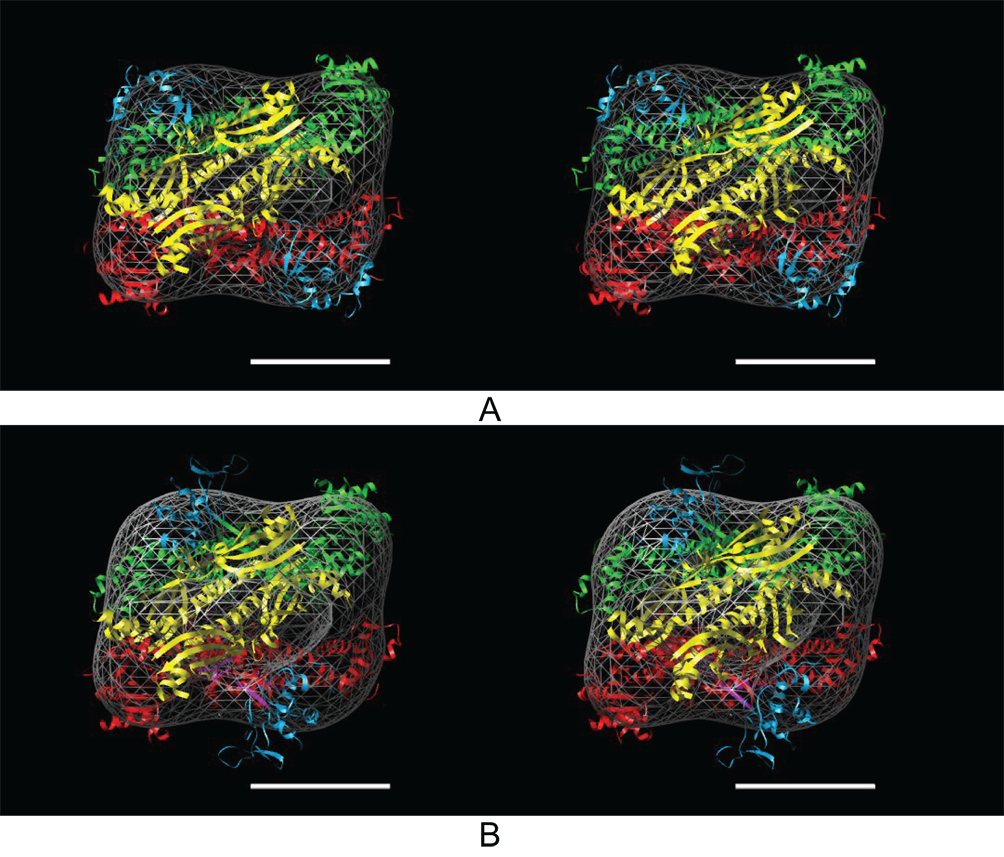

Supplement: Figure S3 — Docking of the 3D EM map of the SecA/SecB complex with the X-ray crystal structures of ecSecA-closed (A) or the ecSecA-open (B), and the X-ray crystal structure of SecB (PDB ID: 1QYN), shown in stereo diagrams. Two SecA protomers are rendered in different colors, green and red. For each SecA protomer, the PBD is in deepskyblue. The SecB tetramer is rendered in yellow. The scale bar represents 5 nm. (TIF) [file pone.0016498.s003.tif]

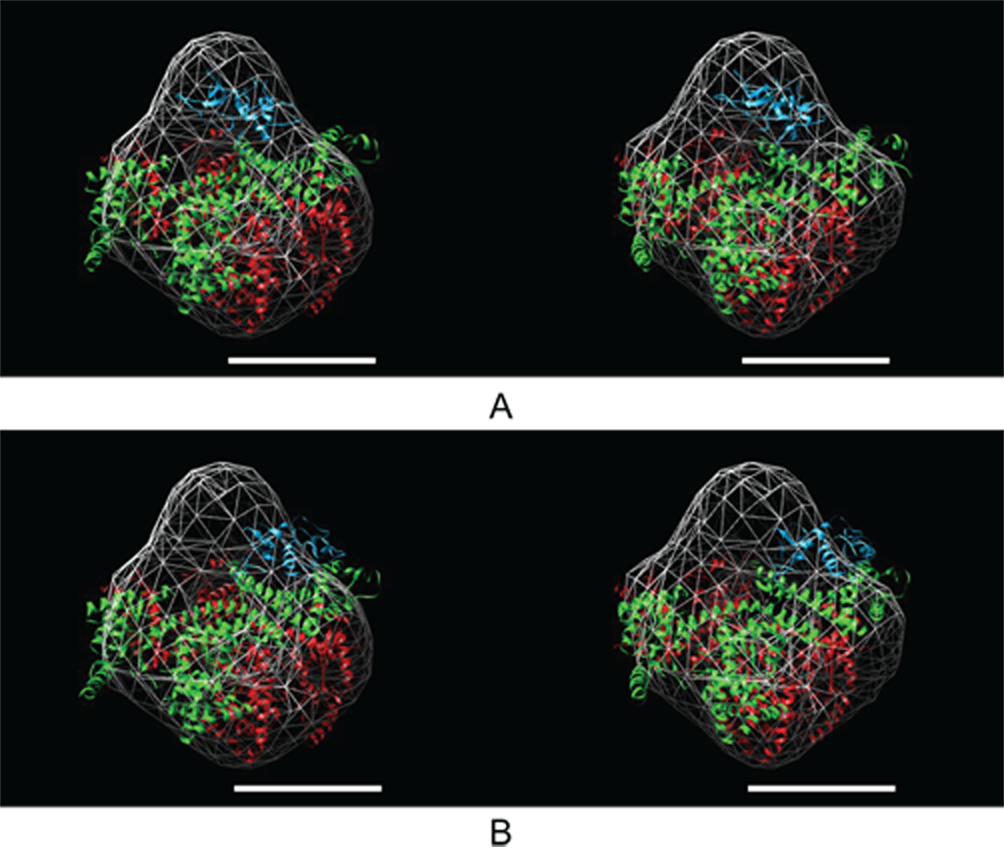

Supplement: Figure S4 — Docking of the X-ray structures of E.coli SecA into the 3D EM map of the SecA/proOmpA/SecB complex, shown in stereo diagrams. One SecA protomer, predominantly binding with SecB/proOmpA, is rendered in green with its PBD domain in deepskyblue. The other one is rendered in red. The red SecA protomer is in the open state (ecSecA-open). (A) The green SecA protomer is in the open state. (B) The green SecA protomer is in the closed state (ecSecA-closed). The scale bar represents 5 nm. (TIF) [file pone.0016498.s004.tif]
